# The Influence of Adhesive Strategy, Type of Dental Composite, and Polishing Time on Marginal Gap Formation in Class I-like Cavities

**DOI:** 10.3390/ma16237411

**Published:** 2023-11-29

**Authors:** Marianna Pires Barbosa, Tiago Braga Rabello, Eduardo Moreira da Silva

**Affiliations:** 1 Analytical Laboratory of Restorative Biomaterials—LABiom-R, School of Dentistry, Universidade Federal Fluminense, Niterói 24020140, Brazil; mariannapiresbarbosa@gmail.com; 2 Department of Dental Clinic, School of Dentistry, Federal University of Rio de Janeiro, Rio de Janeiro 24370110, Brazil; dentistica.ufrj@gmail.com

**Keywords:** dental composite, permanent restorations, bond strategy, dental polishing, gap formation

## Abstract

Even after more than six decades of dental composite invention (1962), there is still controversial information about the time in which composite restorations should be polished in order to avoid marginal gap formation at the tooth–composite interface. The aim of the present study was to analyze the influence of adhesive strategy, the type of dental composite, and polishing time on marginal gap formation (%MG) at the tooth–composite interface. Class I-like cavities were hybridized with a universal adhesive system (Single Bond Universal) through two strategies: selective enamel etching (SEE) or self-etching mode (SEM). Cavities were filled with two types of dental composites: nanofilled (Z350) or bulk fill (Filtek One Bulk Fill—ONE), and polishing was performed immediately or delayed for 7 days (*n* = 5). %MG was evaluated by using a 3D laser confocal microscope. As flexural modulus (FM) and degree of conversion (DC%) are determinants of marginal integrity in dental composite restorations, these properties were evaluated for both composites. Data were analyzed by ANOVA and Tukey’s HSD test (α = 0.05). Cavities hybridized following the SEE strategy presented lower %MG (*p* < 0.05). Z350 showed higher %MG than ONE (*p* < 0.05). There was no difference in %MG between the polishing times when the SEE strategy was used (*p* > 0.05). Z350 presented higher FM than ONE (*p* < 0.05). DC% was found to be not significant (*p* > 0.05). The results suggest that selective enamel etching (SEE) is a better strategy for producing less %MG in composite restorations with enamel margins irrespective of the time in which the restoration is polished.

## 1. Introduction

Due to desirable features such as color mimicry and an on-demand polymerization reaction, dental composites have been widely used in direct esthetic restorations. Irrespective of these clinical advantages, however, these restorative biomaterials still present a question of concern, that is, the volumetric shrinkage that is undergone after their polymerization [1]. During the polymerization reaction, the aliphatic C=C bonds of methacrylate monomers present in the organic matrix of dental composites are converted to unstable C–C bonds that react with each other. This process reduces the intermolecular distances of 0.3–0.4 nm, existing between monomers, to new covalent bonds with 0.15 nm distances. The result of this chain of events is volumetric polymerization shrinkage that ranges from 1% to 6%, depending, among other aspects, on the type of monomers and the volume of filler particles in the composite formulation [2,3]. In dental composite restorations, the competition between the polymerization shrinkage and the bond strength provided by the adhesive system may develop stresses at the cavity walls that might cause deleterious effects such as interfacial damage, the opening of pre-existing or creation of new enamel microcracks, and even the initiation of microcracking within the composite [4,5]. Specifically, at the tooth–restoration interface, shrinkage stress might lead to marginal gap formation [1], microleakage, marginal discoloration, and post-operative sensitivity as well [4]. From a clinical standpoint, these aspects are relevant and may impact the clinical serviceability of dental composite restorations [6].

The extent of shrinkage stress is dependent on the viscoelastic properties of the dental composite. In other words, at a given polymerization shrinkage, the more rigid the dental composite the higher the shrinkage stress. The consequence of this could be an increase in gap formation at the tooth–restoration interface [7]. In this field, previous studies have shown that properties such as the flexural modulus and the degree of conversion may exert an influence on the development of shrinkage stress developed by dental composites [8,9].

Due to specific characteristics such as high translucency, the usage of different photoinitiators from camphorquinone, and the presence of special stress reliever monomers that, according to the manufacturers, allow the development of low shrinkage stress, bulk-fill dental composites have been indicated to be inserted into cavities in single increments up to 6 mm thickness [10]. From one side, this can be seen as a clinical advantage because it may reduce the chairside time, thereby improving the patients’ comfort. Nevertheless, it is well known that large volumes of resin-based materials applied to confined spaces can contribute to the development of interfacial shrinkage stress, which may increase the risk of gap formation [11].

Over the last decade, new developments in the field of dental adhesives have taken place, with universal adhesives being the last evolution in this race [12]. This type of adhesive follows a multi-model concept, allowing its use through different strategies with or without phosphoric acid etching in both dentin and enamel [13]. The first mode is taken as the principal advantage of the universal adhesives because, theoretically, the shallower the demineralization, the lesser the dentin postoperative sensitivity [14]. Nevertheless, there is evidence that the acidic potential of universal adhesives may not be enough to promote proper enamel demineralization [15]. To overcome this drawback, selective etching mode has been used to improve bonding performance in enamel margins. Although previous studies have shown that selective enamel etching is a good strategy when compared to the self-etching strategy [16,17], there is a lack of data regarding the effect of both strategies on marginal gap formation in dental composite restorations. 

The polishing procedure may also influence gap formation at the tooth–restoration interface [18]. The main controversy regarding composite polishing is when to initiate this step. While some manufacturers claim that polishing should be carried out immediately after restoration, several authors have suggested that this procedure should be delayed in order to produce better marginal sealing of the tooth–restoration interface [19]. According to this concept, since the composite polymerization reaction is not completed prior to 24 h and due to the hydrophilic nature of the dental composite’s organic matrix [18], polymerization shrinkage could be partially compensated by the hygroscopic expansion suffered by the material derived from water sorption and subsequent swelling [20]. 

Taking into account that the presence of gaps at the tooth–restoration interface is considered as the first sign of restoration failure [1,21], their identification could contribute to the prognosis of the longevity of composite restorations [22]. Dye-penetration leakage tests and microscopic assessment of the interface have been employed for in vitro detection of interfacial gaps. Nevertheless, these methods are highly subjective and require sectioning of the teeth for analyzing the tooth–restoration interface. These disadvantages encourage the use of more precise technologies for evaluating this response [23]. Therefore, the aim of the present study was to analyze the influence of two polishing times, immediately or after seven days, on gap formation in class I-like cavities restored with two dental composites, nanofilled (Filtek^TM^ Z350) and bulk fill (Filtek^TM^ One Bulk Fill), and one universal adhesive system (Single Bond Universal) applied using two strategies: self-etching mode or selective etching mode, through a 3D laser confocal microscopy analysis. The null hypothesis tested was that there would not be differences in gap formation among all of the experimental groups.

## 2. Materials and Methods

The composition of all materials used in the present study is depicted in Table 1. 

### 2.1. Percentage of Marginal Gap (%MG)

**Figure 1:**
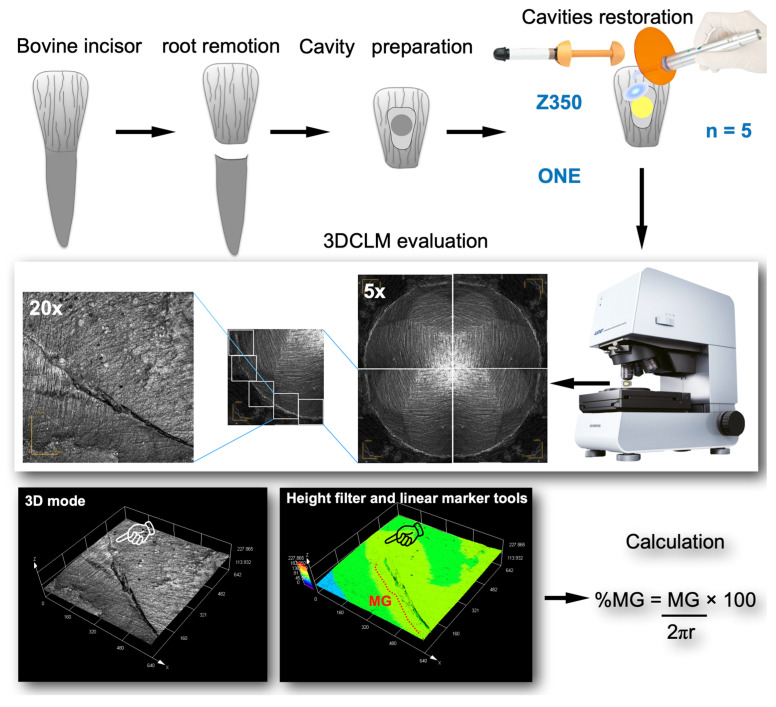
Flow chart for %MG evaluation.

Figure 1 shows the flow chart for %MG evaluation. The buccal surfaces of 40 bovine incisors, stored in 1% aqueous solution of chloramine for two weeks and kept in distilled water at 4.5 °C for less than three months, were wet ground in a polishing machine (DPU 10, Struers, Ballerup, Denmark) using 150-, 400-, 600-, and 1200-grit SiC papers (T223, Saint-Gobain-Norton, Guarulhos, SP, Brazil), for one minute each, until flat enamel surfaces of 10 × 10 mm were produced. Afterwards, the crowns were separated from the roots using a precision cutter machine at 300 rpm (IsoMet^®^ 1000, Isomet, Manassas, VA, USA) and embedded in fast colorless autopolymerizing acrylic resin (JET Classico, Campo Limpo Paulista, SP, Brazil). Cylindrical class I-like cavities (4.0 mm diameter and 1.4 mm depth) were prepared in all flat enamel surfaces using a diamond bur (#3053, KG Sorensen, SP, Brazil) in a high-speed handpiece fixed in a standardized preparation machine (APC 100, Odeme Dental Research, Luzerna, SC, Brazil).

Once prepared, the cavities were bonded with a universal adhesive system (Single Bond Universal, 3M ESPE, St Paul, MN, USA), with half using a self-etching mode (SEM) and the other half using a selective enamel etching mode (SEE). The adhesive system was applied in accordance with the manufacturers’ instructions (Table 2). Then, the cavities were filled using one of the two dental composites. The nanofilled dental composite (Z350) was inserted in two oblique increments using a flat-sided instrument (Suprafill #1, SSWhite, Rio de Janeiro, RJ, Brazil), and the bulk-fill composite (ONE) was inserted in a single increment. The dental composites were photoactivated with an LED unit (Radii-cal, SDI Limited, Bayswater VIC, Australia) using an irradiance of 1200 mW/cm^2^ for 20 s. For each condition, the specimens were divided into two groups according to the time of polishing: immediately or after storage in distilled water for 7 days. Each specimen was wet-polished with 2500- and 4000-grit SiC paper (Corundum Abrasives, Saitama Konosu, Miyamae, Japan) for 1 min using a polishing machine (DPU 10, Struers, Denmark).

The analysis of %MG was carried out using a 3D-laser confocal microscope- 3DLCM (LEXT OLS4001, Olympus, Center Valley, PA, USA) operating on scanning mode XYZ fast scan, with a lens MPLAPONLEXT using a 20× zoom. First, images of each restorative surface were scanned. After that, the images were analyzed using two specific tools of the 3DCLM, a height filter that use color contrast that allowed to identify areas with a gap at the tooth–dental composite interface and a linear marker that allowed to measure the length of the gaps. The %MG was calculated as the ratio of gap length to the entire cavity perimeter by using the following equation:

%MG = (MG/2πr) × 100,

where MG is the length of the tooth–dental composite interfaces with a gap and r is the cylindrical cavity radius.

### 2.2. Flexural Modulus (FM)

The dental composites were applied in a bar-shaped steel split mold (1 × 2 × 10 mm) positioned over a glass plate. After filling the mold to excess, the material surfaces were covered with a polyester strip and glass slide and compressed with a device (500 g) to extrude excess material. The specimens were light cured from the top in two overlapping sections (2 × 1200 mW/cm^2^ for 20 s). The specimen dimensions were recorded using a digital caliper (MPI/E-101, 28 Mitutoyo, Tokyo, Japan). After 24 h storage in distilled water at 37 °C, the specimens were submitted to three-point bending with 6 mm between the supports in a universal testing machine with a load cell of 50 N (DL 10000, Emic, Curitiba, PR, Brazil) at a crosshead speed of 0.5 mm/minute. The flexural modulus (MPa) was calculated from the linear portion of the load/deflection curve using the following equation:

FM = l^3^F/4wh^3^d,

where FM is the flexural modulus, l is the length between the supports, F is the applied load, w is the width of the specimen, h is the height of the specimen, and d is the deflection at load F. Ten specimens were produced from each resin composite.

### 2.3. Degree of Conversion (DC%)

Standardized increments of each dental composite (*n* = 5) were positioned on an ATR crystal of the FT-IR spectrometer (Alpha-P/Platinum ATR Module, Bruker Optics, Ettlingen, Germany). The spectra of uncured increments were obtained in the range between 1500 and 1800 cm^−1^, operating with 40 scans at 4 cm^−1^ resolution. After photoactivation of each increment, the spectra were recorded again using the same parameters. For Z350, the DC% was calculated from the ratio between the integrated area of absorption bands of the aliphatic C=C bond (1639 cm^−1^) to that of the aromatic C=C bond (1609 cm^−1^), used as an internal standard, which were obtained from the cured and uncured increments. For ONE, which has no aromatic C=C bonds, the integrated area of the carbonyl >C=O bond (1720 cm^−1^) was used as the internal standard. The following equation was used:

DC% = 100 × [ 1 − (R cured/R uncured)]

where R = integrated area of absorption 1639 cm^−1^ or 1720 cm^−1^ bond/integrated area of absorption 1609 cm^−1^ bond

### 2.4. Statistical Analysis

The statistical analysis was performed using Statgraphics 5.1 software (Manugistics, Rockville, MD, USA). Initially, the normal distribution of errors and the homogeneity of variances were checked by Shapiro–Wilk’s test and Levene’s test. Based on these preliminary analyses, the flexural modulus and degree of conversion data were analyzed by one-way ANOVA and Tukey’s HSD post hoc test, and gap formation data were analyzed by three-way ANOVA and Tukey’s HSD post hoc test. All statistical analyses were performed at a significance level of α = 0.05.

## 3. Results

### 3.1. Percentage of Marginal Gap (%MG)

**Figure 2:**
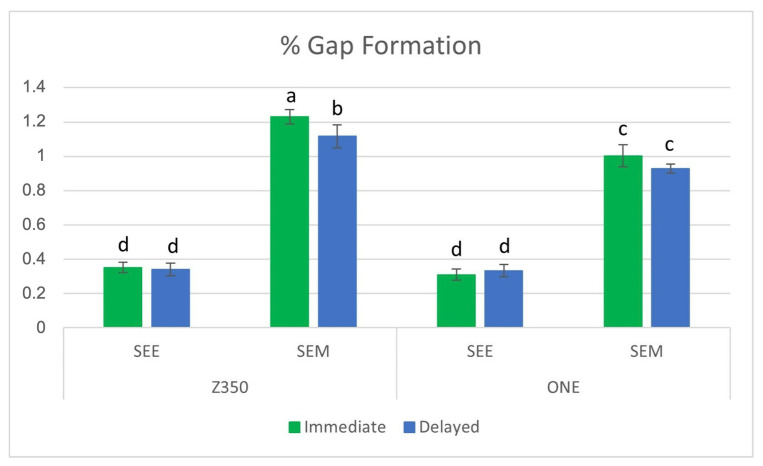
Mean and standard deviation of %MG. Bars with the same letter are statistically similar (*p* > 0.05).

**Figure 3:**
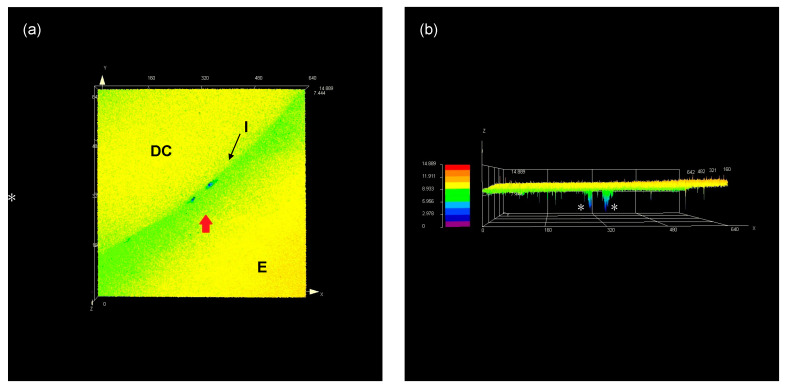
Representative 3Dlaser confocal microscope images used for %MG evaluation. Top view (**a**) red arrow shows gaps at the tooth–composite interface. 3D view (**b**) white asterisks show the depth of the points marked with red arrow in (**a**). DC: dental composite; E: enamel; I: tooth–composite interface.

The results for the %MG are shown in Figure 2. Three-way ANOVA showed statistical significance for the three independent factors (adhesive system, dental composites, and polishing time), as well as for the three-way interaction. Regarding the dental composites, Z350 showed higher %MG than ONE (*p* < 0.05). For the adhesive system, %MG was statistically higher for cavities restored using the SEM strategy (*p* < 0.05). With respect to polishing time, the statistical significance was found only for the groups submitted to the self-etching-mode strategy (*p* < 0.05). Contrarily, for selective enamel etching, no difference in %MG was found between both polishing times (*p* > 0.05). Figure 3 shows representative 3D-laser confocal microscope images used for %MG evaluation. The red arrow shows points with a gap at the tooth–composite interface (a). The white asterisks show the depth of these points in a 3D view (b).

### 3.2. Flexural Modulus (FM)

**Figure 4:**
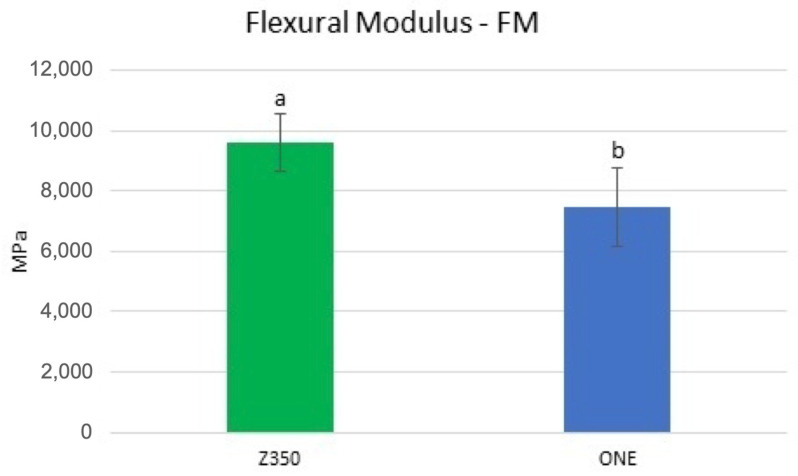
Mean and standard deviation of the flexural modulus (MPa) for the two dental composites. Different letters show statistical significance (Tukey’s HSD test, *p* < 0.05).

The results for the FM are presented in Figure 4. Tukey’s HSD test showed that Z350 presented a significantly higher flexural modulus than ONE (*p* < 0.05).

### 3.3. Degree of Conversion (DC%)

**Figure 5:**
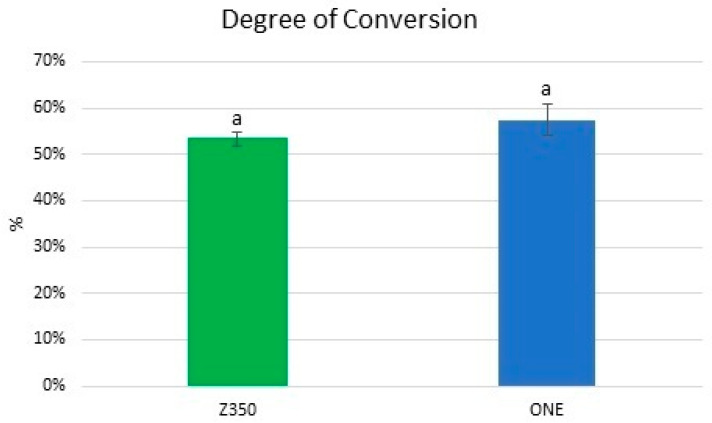
Mean and standard deviation of the degree of conversion (%) for all dental composites. Bars with the same letter are statistically similar (*p* > 0.05).

The results for the DC% are displayed in Figure 5. No significant difference was observed between the dental composites (Tukey’s HSD test, *p* > 0.05).

## 4. Discussion

Marginal adaptation has been described as one of the most important factors that influences the clinical outcome of a dental composite restoration [24]. Moreover, even considering that the clinical protocol to build up dental composite restorations is well accepted by clinicians, there is still no consensus regarding the best time in which the steps of finishing and polishing of these restorations must be done [1,19,20]. Therefore, the present study focused on the influence of polishing time (immediate or delayed by 7 days) on gap formation at the tooth–dental composite interface. The choice of the independent variables evaluated here was thought in an attempt to cover all possibilities used in daily clinical practice. Filtek Z350 XT is a nanofilled dental composite that, according to its manufacturer (3M ESPE), must be applied using the incremental technique. On the other hand, Filtek One Bulk Fill is representative of bulk-fill dental composites indicated to be applied in single increments up to 5 mm. The two bonding strategies, selective enamel etching (SEE) and self-etching mode (SEM), were employed because they are well established in the literature. Finally, the polishing time (immediately and after 7 days) was the main target focused on here.

As the three independent factors (adhesive strategy, type of dental composite, and polishing time) influenced the gap formation at the tooth–dental composite interface, the null hypothesis of the present study was rejected. Although previous studies have been concerned with assessing the gap width [25,26], the presence of marginal gaps at the tooth–dental composite interface per se could be taken as the first signal for allowing the establishment of microleakage phenomenon at the tooth–dental composite interfaces [1]. In other words, regardless of their widths, the simple presence of gaps represents an open door to oral fluids and bacteria that might start the degradation process of the restoration interface. In addition, it is more reliable to evaluate the marginal gap length along the restoration margins because a reduced gap length might represent fewer margins that are prone to degradation [1]. Therefore, similar to previous studies [1,4,11,23], in the present study, the percentage of the gap length along the total length of the cavity interface was measured.

Due to the limitations inherent to the applied methods, the evaluation of marginal gaps remains as a challenge for in vitro investigations [27]. Even powerful tools such as scanning electron microscopy (SEM) and X-ray microcomputed tomography (μ-CT) present some disadvantages that can impair or introduce bias to evaluation [28]. For example, for SEM, analysis using 2D images, there is the dependency on operator ability and the impossibility of measuring the gap volume and depth [29]. For μ-CT, the materials’ radiopacity may impact gap visualization through software reconstruction [30]. In the present study, %MG was measured through 3D laser confocal microscopy (Figure 1). The used equipment is a noncontact laser scanning microscope that allows for the fast acquisition and non-destructive observation of images under XY resolution of 0.12 μm and a 10 nm resolution in the *Z*-axis. In the present study, a height filter tool was used that employs a color scale to determine the depth of the gaps at the tooth–dental composite interface, and a linear marker was used that allowed for measuring the length of these gaps through the entire interface. This approach is capable of carrying out measurements with high accuracy and repeatability, probably avoiding the introduction of bias to this kind of evaluation. Figure 1 clearly shows the absence of a gap in the image where the height filter tool was used (black pointers). Contrarily, this region could have been appointed as a gap in the black–white counterpart images (white pointer). These aspects may reinforce the suitability of this method for precisely evaluating marginal gaps in dental composite restorations [31].

The primary aim of a dental adhesive is to provide retention of the composite to the cavity walls, withstanding the stresses derived from dental composite polymerization shrinkage. This aspect is pivotal for less gap formation at the tooth–dental composite interface [32]. Thanks to the possibility to be applied through different bonding strategies, e.g., etch-and-rinse and self-etch mode, universal dental adhesives have become more popular among clinicians. This type of adhesive has a phosphoric acid ester in its composition, such as MDP in Single Bond Universal, which is, theoretically, capable of demineralizing both dentin and enamel [33]. Nevertheless, there is strong evidence that an additional step involving selective etching of the cavity margins, prior to applying the universal adhesive, may improve the bond strength to the enamel [33].

Irrespective of the type of composite and time of polishing, the cavities treated with the SEE strategy presented lower %MG than those treated with the SEM strategy (Figure 2). These findings suggest that the lower acidic potential of universal adhesives is less effective to demineralize the high mineral phase of enamel when compared to phosphoric acid [33,34,35]. Reinforcing this concept, Sender et al. (2020) [15] showed, through topographic analysis using confocal Raman spectroscopy, that enamel surfaces treated with Scotchbond Universal (MDP-containing) presented a smooth and homogeneous surface, with regular levels in the concentration of Hap when compared with surfaces produced by phosphoric acid in different concentrations (35% and 37%). According to the authors, the finding is indicative of MDP (a phosphoric acid ester, pH of 2.7) not being capable of fully demineralizing enamel for creating an adequate etching pattern for optimizing the bond strength when compared with phosphoric acid-etching.

In the present study, %MG was higher in cavities restored with Z350. This finding can be explained by the highest flexural modulus presented by this dental composite (Figure 4). Flexural modulus is a property directly related to shrinkage stress, a phenomenon that might be explained through a parallel to Hooke’s law [15], in which shrinkage stress is the product of polymerization shrinkage versus the flexural modulus. According to this law, there is a significant relationship between shrinkage stress and the flexural modulus, rather than between polymerization shrinkage and shrinkage stress [36]. Based on data supplied by the manufacturers (Table 1), Z350 has 63.3 vol% of fillers while ONE has only 58.5 vol%. Although a higher filler content is related to a possible reduction in the volumetric shrinkage, this can also increase the flexural modulus of a dental composite [37,38]. This aspect might be used to explain the higher flexural modulus presented by Z350, which, in turn, could have influenced the higher %MG developed by this dental composite. Beside the lower flexural modulus, some aspects related to the organic matrix of ONE might also have contributed to the lower %MG presented by this composite. ONE has a high-molecular-weight aromatic urethane dimethacrylate (AUDMA) that, according to its manufacturer, helps to moderate the volumetric shrinkage as well as the stiffness of its polymeric phase, features that may contribute to decreasing the polymerization stress developed by the composite. In addition, during polymerization, AFM, a type of addition–fragmentation monomer, reacts into the developing polymer as with any methacrylate, including the formation of cross-links between adjacent polymer chains. Moreover, AFM contains a third reactive site that cleaves through a fragmentation process during polymerization. These processes provide a mechanism for the relaxation of the polymer network with subsequent stress relief [38]. These aspects were confirmed by a previous study showing that AFM contributed to lower shrinkage stress due to direct stress relief through the addition–fragmentation chain transfer process [39].

A deeper interpretation of Figure 2 allows interesting comments regarding the influence of polishing time on %MG. When cavities were treated with the SEE strategy, neither Z350 nor ONE presented difference in %MG between immediate and delayed polishing. This aspect suggest that the improved bond strength promoted by the SEE strategy overshadowed the influence of specific aspects related to each composite, i.e., the flexural modulus for Z350 and the organic matrix of ONE on %MG. Based on this, it is reasonable to claim that the time of polishing might not be relevant when the SEE strategy is used. This thought reinforces once more the effectiveness of this bond strategy for avoiding marginal gap formation at the tooth–composite interface. On the other hand, Figure 2 shows that Z350 presented statistically lower %MG after 7 days (delayed polishing). Evaluating the salivary sorption of dimethacrylate-based polymeric matrixes, Gonçalves et al. [40] showed that the increase in TEGDMA content increased the salivary sorption of binary matrixes. The authors explained this finding through a high level of hydrogen bonding between ether linkages in the TEGDMA molecule and water and because of the primary cyclization inherent to TEGDMA flexibility. According to the authors, cyclization might create nanopores to which water diffuses without establishing hydrogen bonds with the polar groups of TEGDMA. Thus, one can suggest that the lower %MG presented by Z350 after 7 days of polishing was due to a water sorption increase derived from the presence of TEGDMA in its composition. This possibility is supported by previous studies showing that hygroscopic expansion is the main mechanism for shrinkage stress compensation in dental composites [41,42].

Although the present study added interesting aspects regarding the effect of the adhesive strategy, the type of dental composite, and polishing time on marginal gap formation at the tooth–composite interface, limitations such as using only two dental composites and the absence of an internal adaption evaluation still exist. These aspects should be addressed in future investigations.

## 5. Conclusions

Within the limitations of the present study, it was concluded that, irrespective of the time in which the restoration is polished, selective enamel etching (SEE) could be seen as a better strategy for producing fewer marginal gaps in dental composite restorations.

## Figures and Tables

**Table 1:** Overview of the materials and their compositions used in this study.

Material (Manufacturer)	Type	Shade	*Composition
**Filtek™ Z350 XT (3M ESPE, St Paul, MN, USA)**	Nanofilled dental composite	A3	Zirconia/silica (78.5% wt%, 63.3 vol%) Bis-GMA, UDMA, TEGDMA, and Bis-EMA, initiators, and stabilizers.
**Filtek™ One Bulk Fill (3M ESPE, St Paul, MN, USA)**	Bulk-fill dental composite	A3	Zirconia/silica and an ytterbium trifluoride filler consisting of agglomerate 100 nm particles (76.5% wt%, 58.5 vol%), AFM (dynamic stress-relieving monomer), AUDMA, UDMA and 1, 12-dodecane-dimethacrylate, initiators, and stabilizers.
**Single Bond Universal (3M ESPE, St Paul, MN, USA)**	Universal adhesive	---	MDP phosphate monomer, dimethacrylate resins, HEMA, 3M™ Vitrebond™ copolymer, filler, ethanol, water, initiators, and silane.

* According to safety data sheets and instructions for use from the respective manufacturers. Bis-GMA, bisphenolglycidil methacrylate; UDMA, urethane dimethacrylate; TEGDMA, triethyleneglycol dimethacrylate; Bis-EMA, ethoxylated bisphenol A glycol dimethacrylate; AUDMA, aromatic urethane dimethacrylate, HEMA, 2-hydroxiethil methacrylate; MDP, methacryloyloxydecyl dihydrogen phosphate.

**Table 2:** Adhesive system manufacturers’ instructions.

Material (Manufacturer)	Etch	Adhesive	Light Cure
**Single Bond Universal (3M ESPE, St Paul, MN, USA)**	---	Rub the adhesive in for 20 s; 5 s of gentle air.	10 s light cure.
**Single Bond Universal (3M ESPE, St Paul, MN, USA)**	15 s selective enamel etching; 30 s rinse; blot excess water using absorbent paper.	Rub the adhesive in for 20 s; 5 s of gentle air.	10 s light cure.

## Data Availability

The data that support the findings of this study are available from the corresponding author upon request.
